# Supplementary material for: Calcification of Various Bioprosthetic Materials in Rats: Is It Really Different?
Source: Int J Mol Sci. 2023 Apr 14;24(8):7274. doi: 10.3390/ijms24087274 (PMC10139218; doi:10.3390/ijms24087274)
Supplement: Supplementary file 1 [file ijms-24-07274-s001.zip › ijms-2319492-supplementary.docx]

SUPPORTING INFORMATION (additional explanatory figures)

CALCIFICATION OF VARIOUS BIOPROSTHETIC MATERIALS IN RATS: IS IT REALLY DIFFERENT?

Irina Y.Zhuravleva^1*^, Elena V. Karpova^2^, Anna A. Dokuchaeva^1^, Anatoly T.Titov^3^, Tatiana P. Timchenko^1^, Maria B.Vasilieva^1^


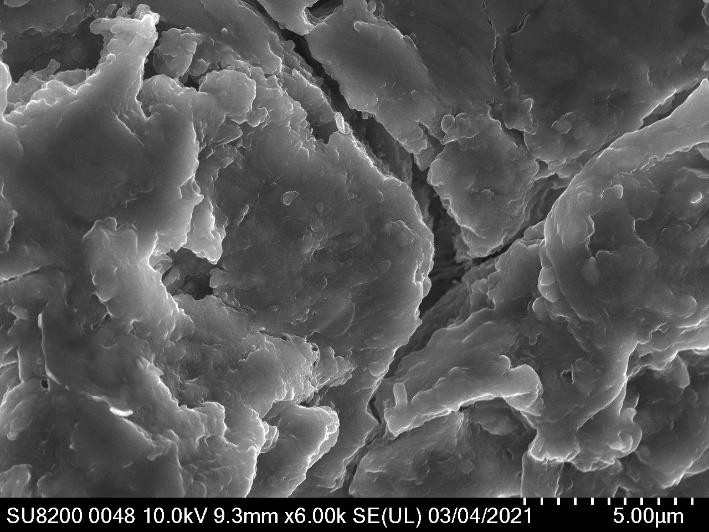

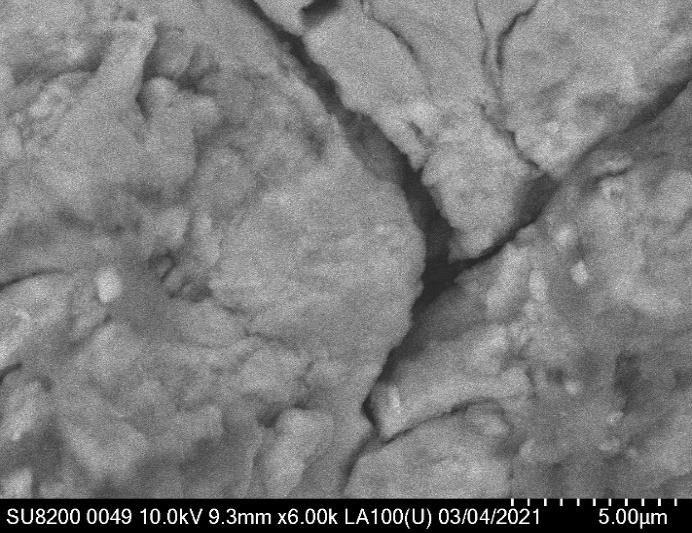

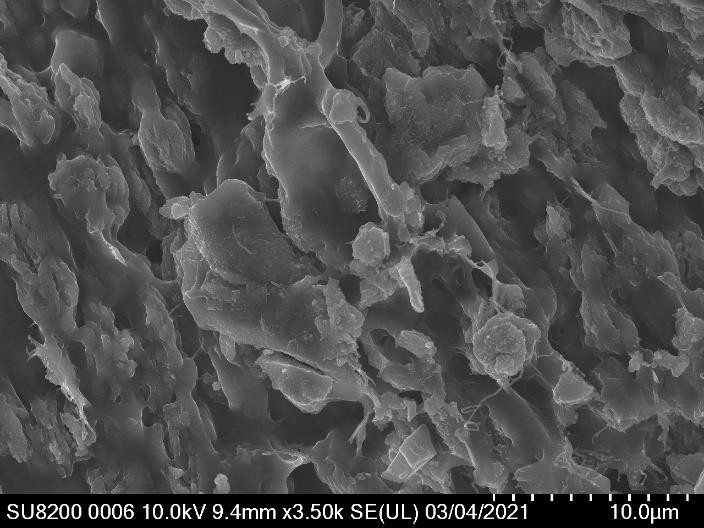

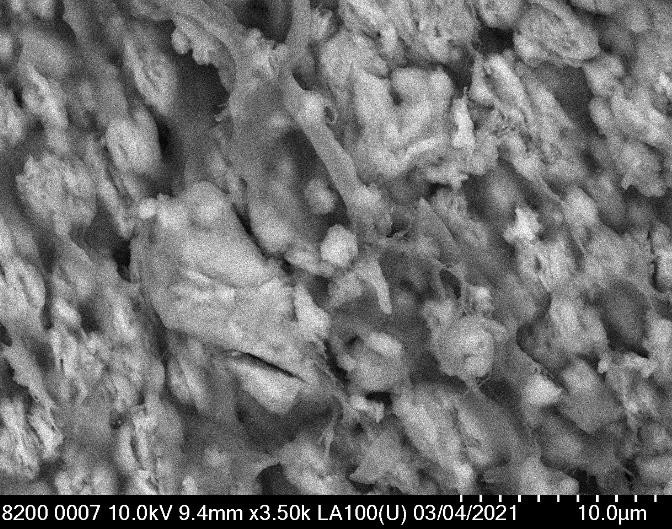


**A**

**B**

**C**

**D**

**Figure S1.** Calcified structure verification. Images of Ao-GA (A, B) and Ao-DE (C,D). White structures at back scattering electron images are calcium phosphate deposits (B, D). In the yellow rectangles (A, C) are the areas shown at high resolution in Figure. 5.


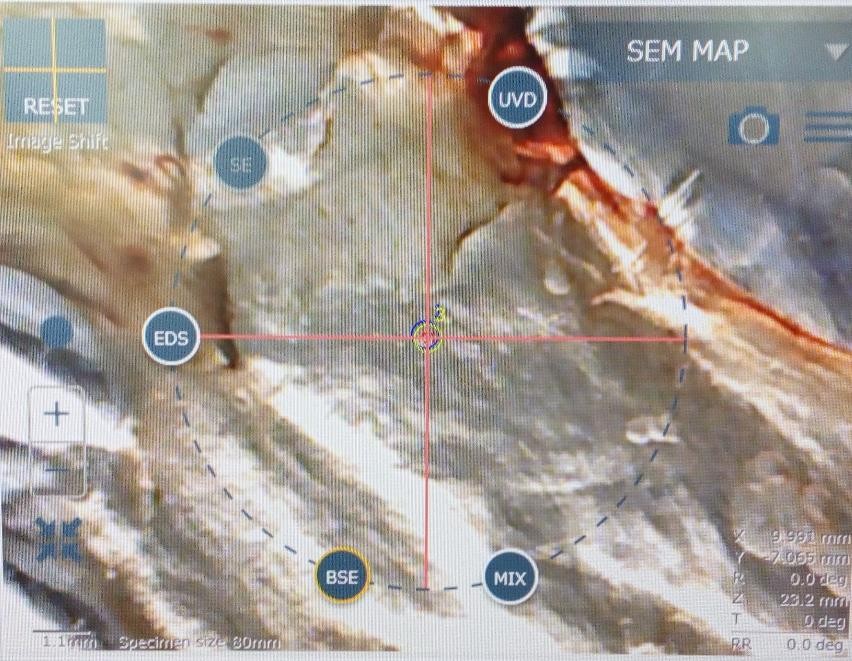


**Figure S2.** Studying of the dried fascial tissue by SEM. The sample is placed on the sample holder.

**Figure S3.** Fresh bovine jugular vein samples obtained from two animals. Mallory stain; scale bars 100 μm.


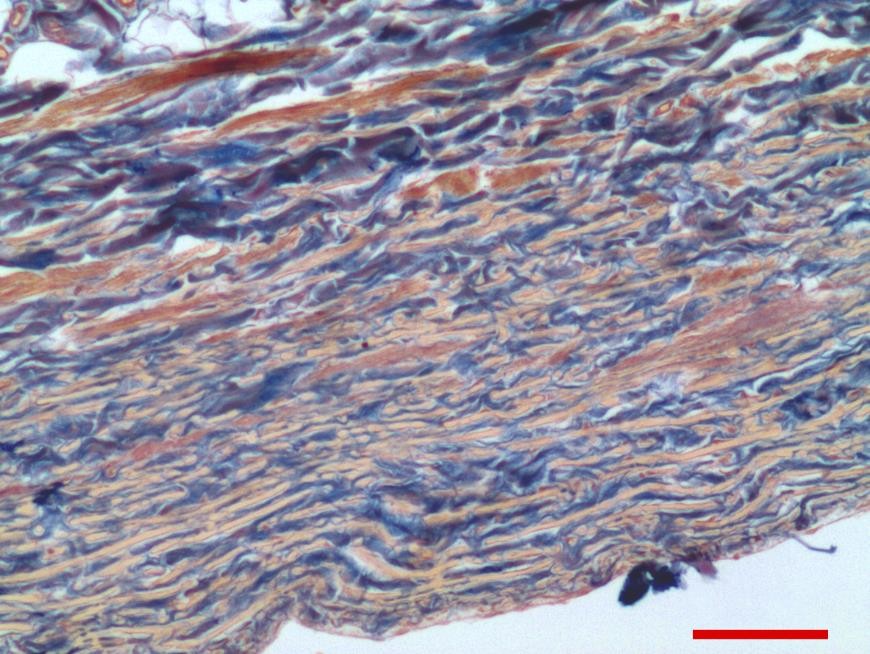

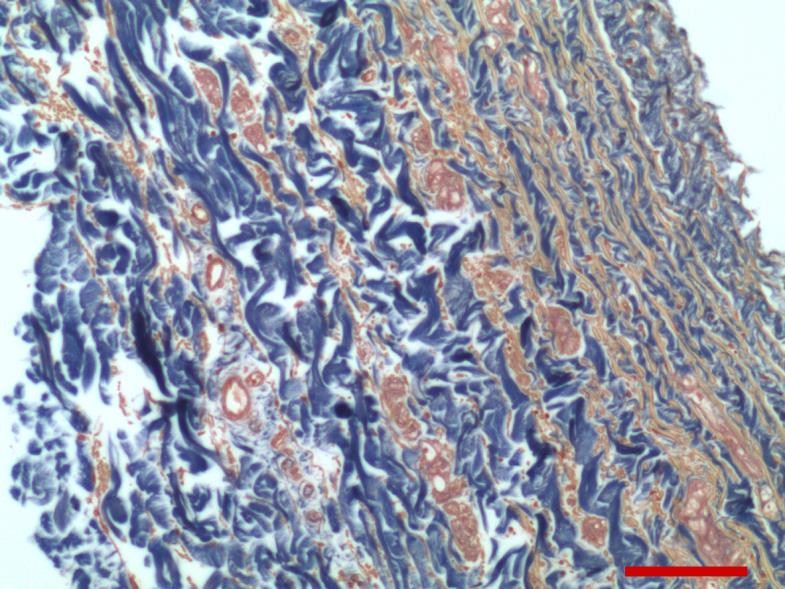

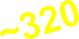

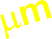

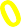

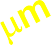


**b**

**a**

Elastin fibers are yellow. With approximately the same wall thickness, the thicknesses of the elastin fibers’ layer differ by almost 2 times (~320 μm (a) and ~140 μm (b)).


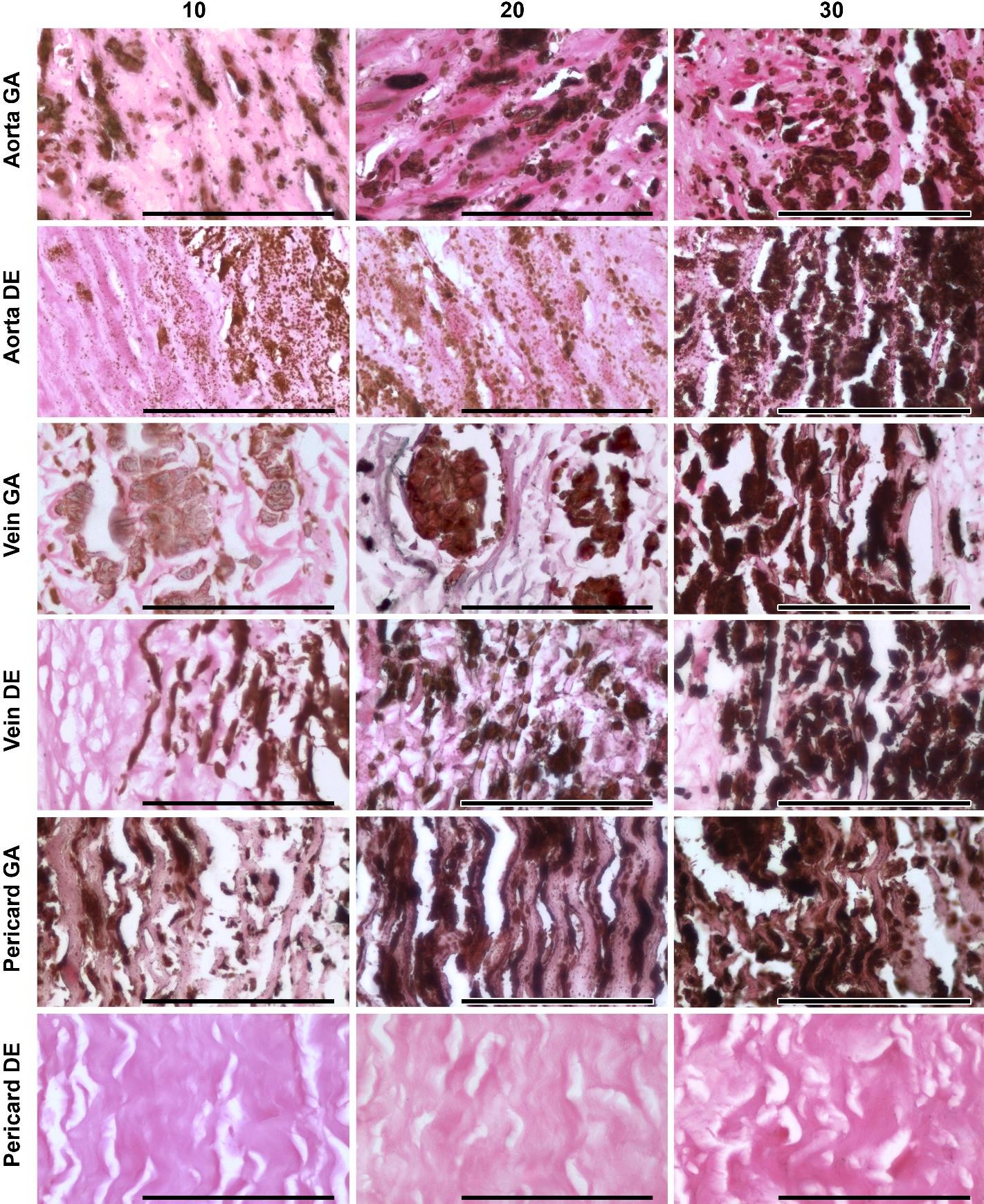


**Figure S4.** Calcium phosphate deposits in biomaterials 10, 20 and 30 days after implantation. No sign of calcification in DE-Pe. Scale bars 100 μm.


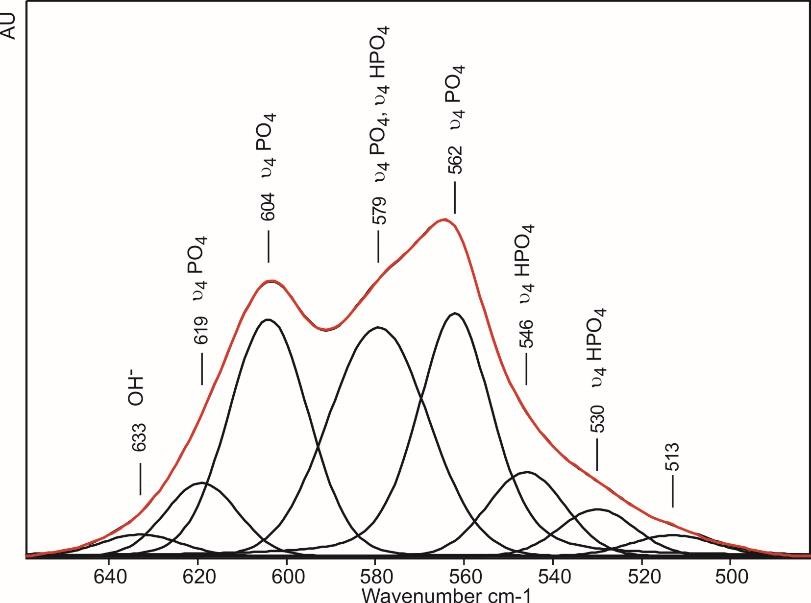


**Figure S5.** Spectral profile of the difference between the GA-Pe spectra 30 days after implantation in a rat and the initial GA-Pe in the range of 650 - 450 cm^-1^.
